# The utility of ESR, CRP and platelets in the diagnosis of GCA

**DOI:** 10.1186/s41927-019-0061-z

**Published:** 2019-04-10

**Authors:** Fiona Li Ying Chan, Susan Lester, Samuel Lawrence Whittle, Catherine Louise Hill

**Affiliations:** The Rheumatology Department, The Queen Elizabeth Hospital, 28 Woodville Road, Woodville, SA 5011 Australia

**Keywords:** Giant cell arteritis, Inflammatory markers, Diagnosis, Vasculitis

## Abstract

**Background:**

To compare the utility of ESR, CRP and platelets for the diagnosis of GCA.

**Method:**

A clinical diagnosis of GCA was determined by case-note review of 270 individuals (68% female, mean age 72 years) referred to a central pathology service for a temporal artery biopsy between 2011 and 2014. The highest levels of ESR, CRP and platelets (within 2 weeks of diagnosis) were documented. Evaluation of ESR, CRP and platelets for the diagnosis of GCA were compared using Receiver Operating Characteristic Area Under the Curve (ROC-AUC), and sensitivity/specificity at optimum cut-off values.

**Results:**

GCA was clinically diagnosed in 139 (67%) patients, with 81 TAB positive. The AUC estimates for ESR, CRP and platelets were comparable (0.65 vs 0.72 vs 0.72, *p* = 0.08). The estimated optimal cut-off levels were confirmed at 50 mm/hour for ESR, and determined as 20 mg/L for CRP and 300 × 10^9^/L for platelets. Sensitivity estimates for these three tests were comparable (*p* = 0.45) and ranged between 66% for ESR and 71% for platelets. Specificity estimates were also comparable (*p* = 0.11) and ranged between 57% for ESR and 68% for CRP. There was only moderate agreement between the three positive tests (agreement 67%, kappa: 0.34), and when considered collectively, CRP and platelet positive tests were independent predictors of GCA (*p* <  0.001), but the ESR was not (*p* = 0.76).

**Conclusion:**

ESR, CRP and platelets are moderate, equivalent diagnostic tests for GCA, but may yield disparate results in individual patients. A combination of CRP and platelet tests may provide the best diagnostic utility for GCA.

## Background

Giant cell arteritis (GCA) is a vasculitis of large and medium-sized vessels and is considered he most common form of vasculitis in the white population over the age of 50 [1] with official descriptions present since 1932 [2]. Temporal artery biopsy remains the gold standard for diagnosis [3, 4] but has limited sensitivity due to the segmental nature of this disease. The sensitivity rates also vary according to the cranial or large-vessel phenotypes of GCA. Rapid diagnosis and management is paramount in GCA due to its potential to cause irreversible vision loss [5].

The American College of Rheumatology research classification criteria for GCA requires three or more of the following five criteria [6]: Age 50 years and older, new onset of localized headache, temporal artery tenderness on palpation or decreased pulsation, an abnormal temporal artery biopsy or an erythrocyte sedimentation rate (ESR) of 50 mm/h or more. However, in recent years alternative acute phase reactants such as C-reactive protein (CRP) and platelets have been proposed as more sensitive markers in the diagnosis of GCA. The postulated mechanism of thrombocytosis in promoting inflammation stems from their early interaction with the endothelium in inflammatory states during which they provide adhesion molecules and chemotactic stimulation to aid in the recruitment of leukocytes and enhance the release of different proinflammatory mediators [7]. Despite advances in our understanding, there continues to be a lack of specific diagnostic markers in the diagnosis of GCA, which pose a significant challenge, especially when there is a discrepancy between inflammatory markers.

Hence, the purpose of this study was to review the utility of ESR, CRP and platelet count in the initial diagnostic process for GCA to aid in clinical situations where there is a discordance between the laboratory results.

## Methods

We performed a retrospective audit of all temporal artery biopsies reviewed at South Australian teaching hospitals from January 1st, 2011 to December 31st, 2014. A structured case note review was undertaken of both electronic and paper medical records. The highest recorded values for ESR, CRP and platelet count within a two-week period prior to biopsy were recorded from Oacis (South Australian state-wide electronic medical record system) and from physician documentation in paper medical records. The two-week period was determined to be optimal by taking into account the administrative and clinical delays associated with the organisation of a temporal artery biopsy. TAB results, with no review of actual specimens and a final clinical diagnosis (irrespective of biopsy results) were also noted. Final clinical diagnosis was at the discretion of the treating physician and in biopsy negative cases these were made based upon suggestive clinical features and clinical response to glucocorticoid therapy. The diagnosis was reviewed after treatment and follow up period of at least 3 months.

Patients were excluded when one or more laboratory data (ESR, CRP or platelet count) could not be collected due to either results being inaccessible (due to alternative laboratories in rural or private healthcare referrals), or not being performed due to physician preference. Patients with no record of a final clinical diagnosis due to a lack of follow up data were also excluded. Reasons for lack of follow up data included departure of non-domestic patients, alternative non-rheumatological diagnosis and limited access to private and rural healthcare medical records where patients were subsequently reviewed.

Statistical analysis was performed in Stata v14 (StataCorp LLC, Texas, USA). The performance of ESR, CRP and platelet counts as diagnostic tests for GCA was analysed using Receiver Operating Characteristic (ROC) analysis, performed using non-parametric ROC regression, with 5000 bootstrap replicates. Optimum cut-off values to define a positive test were estimated at the maximum of the product of the sensitivity and specificity (Liu’s method). Generalized McNemar tests were used to compare positive/negative results for ESR, CRP and platelet tests matched within each individual, and prevalence and bias adjusted kappa was used to quantify agreement between the three test results. The three-way relationship between ESR, CRP and platelet positive tests for the prediction of GCA were evaluated by multi-variable logistic regression.

## Results

**Fig. 1:**
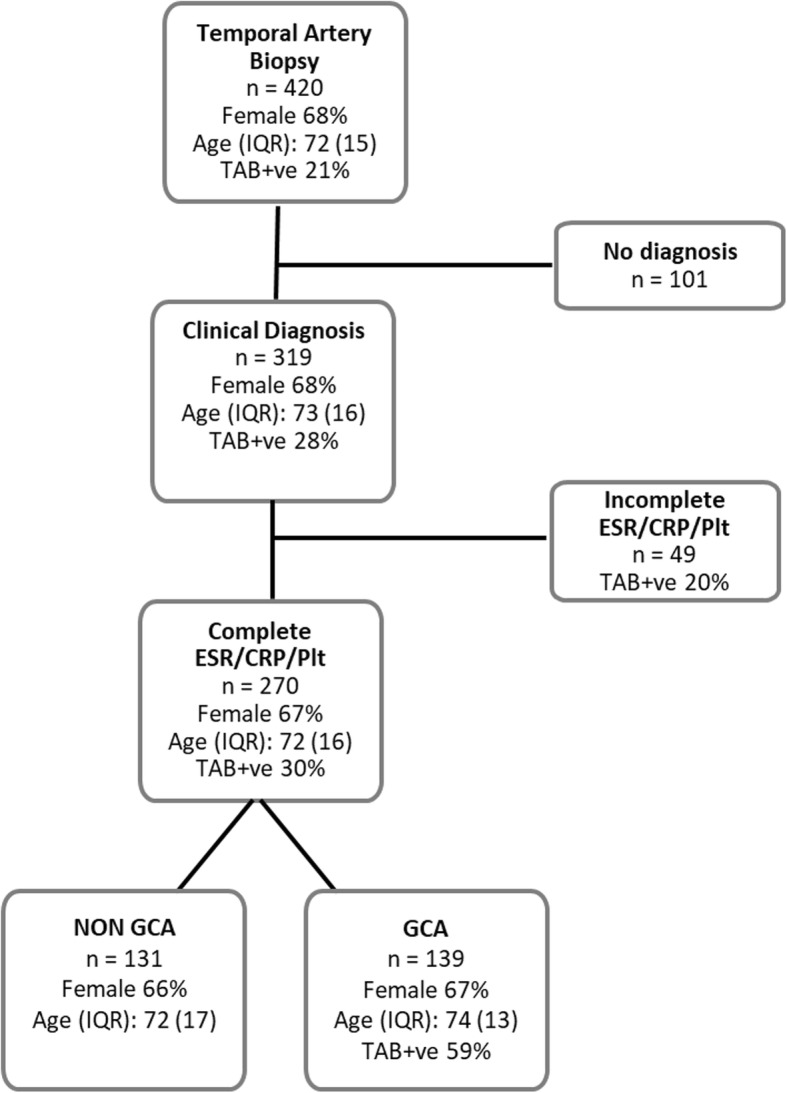
Study Flowchart

A total of 420 medical records of patients referred for a temporal artery biopsy (TAB) were reviewed with 101 excluded due to incomplete follow-up data and further 49 excluded due to incomplete laboratory data (Fig. 1). Therefore, a total of 270 patients were included in the analysis. There was no difference in age or gender between patients who were included (*n* = 270) or excluded (*n* = 150) (Fig. 1).

Of the 270 included patients, 139 (51%) received a physician diagnosis of GCA, with a positive TAB reported for 81/139 (58%). A negative TAB result was reported for 57 GCA patients and one TAB result was inconclusive.

**Fig. 2:**
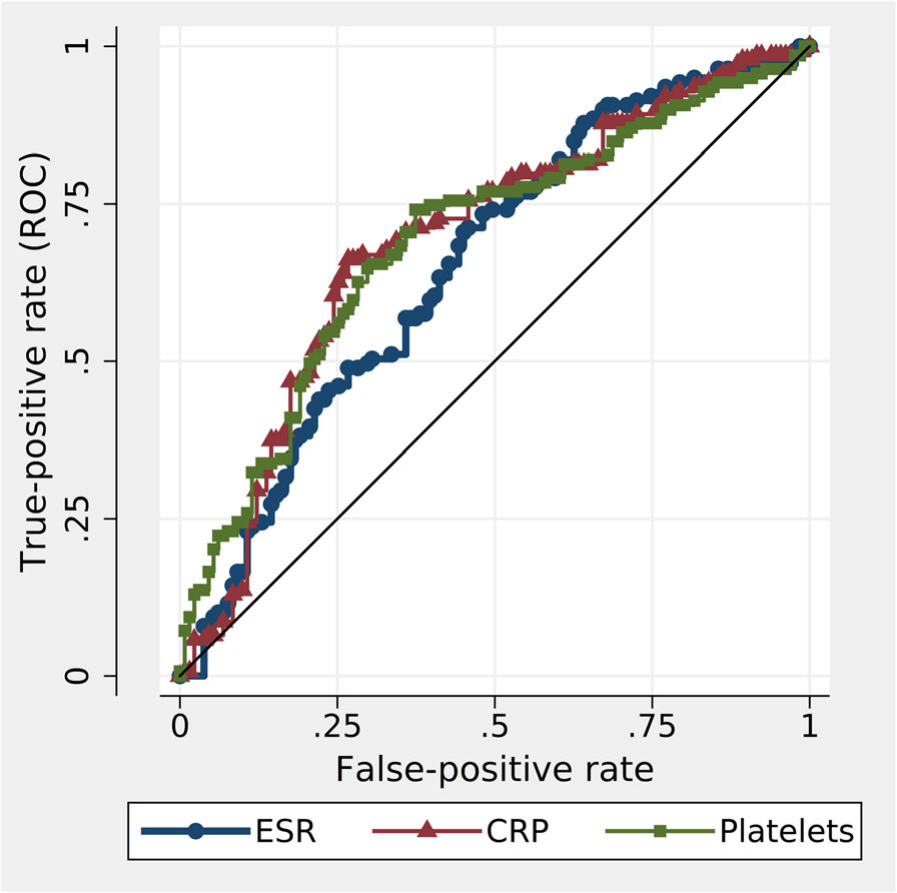
Receiver operating curves (ROC) analysis to compare the diagnostic utility of ESR, CRP and platelet counts for the diagnosis of GCA. The three ROC curves are not significantly different (*p* = 0.08)

ROC curves (Fig. 2) were used to compare the diagnostic utility of ESR, CRP and platelet counts for GCA, and area under the curve (AUC) estimates are reported in Table 1. While the AUC estimate for the ESR (0.65) is slightly less than for CRP (0.72) or platelet counts (0.72), indicating a slightly lower utility of the ESR, the three ROC curves are in fact comparable (*p* = 0.08), and AUC values in this range indicate only moderate, or borderline acceptable performance as diagnostic tests [8].

**Table 1:** Receiver Operating Characteristic (ROC) Area under the Curve (AUC) estimates, cut-off estimates to define a positive test, and diagnostic accuracy of positive tests for ESR, CRP and Platelets for a diagnosis of GCA. Numbers in brackets represent 95% confidence intervals

	ESR (mm/hr)	CRP (mg/L)	Platelets (10^9^/L)
AUC	0.65 (0.57, 0.72)	0.72 (0.65, 0.79)	0.72 (0.65, 0.79)
Estimated cut-off^a^			
Bx- GCA vs non-GCA	44 (21, 66)	23 (12, 35)	319 (291, 347)
Bx + GCA vs non-GCA	47 (28, 65)	23 (16, 32)	297 (263, 330)
All GCA vs non-GCA	47 (28–65)	23 (19, 28)	297 (272, 321)
Selected cut-off	50	20	300
Proportion positive at cut-off (%)			
non-GCA (*n* = 131)	56 (43%)	42 (32%)	49 (37%)
GCA (*n* = 139)	91 (65%)	93 (67%)	99 (71%)
All (*n* = 270)	147 (54%)	135 (50%)	148 (55%)
Diagnostic accuracy of positive tests			
Sensitivity (%)	65.5 (56.9, 73.3)	66.9 (58.4, 74.6)	71.2 (62.9, 78.6)
Specificity (%)	57.3 (48.3, 65.9)	67.9 (59.2, 75.8)	62.6 (53.7, 70.9)
Positive Predictive Value (%)	61.9 (53.5. 69.8)	68.9 (60.4, 76.6)	66.9 (58.7, 74.4)
Negative Predictive Value (%)	61.0 (51.8, 69.6)	65.9 (57.3, 73.9)	67.2 (58.1, 75.4)
Correct (%)	61.4	67.4	67.0

^a^Cut-off values were determined at the maximum of the product of the sensitivity and specificity (Liu’s method)

**Fig. 3:**
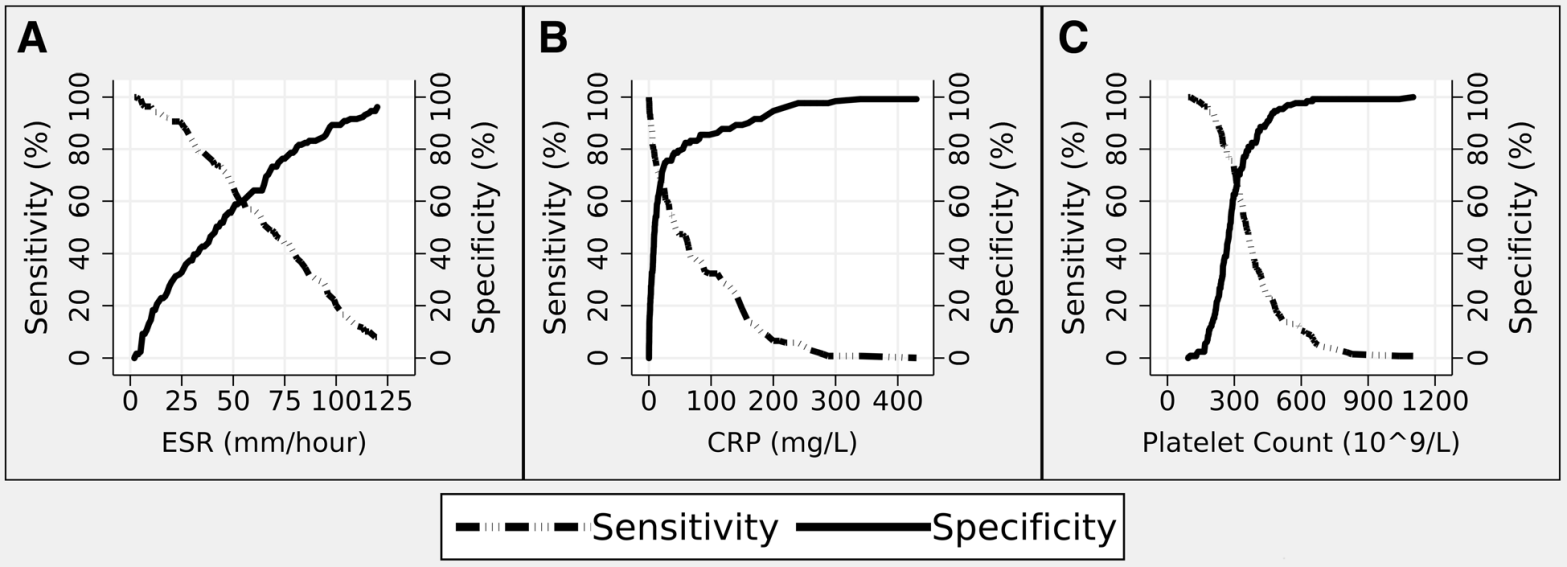
Sensitivity and Specificity curves for different cut-off values of **a** ESR, **b** CRP and **c** Platelet counts for the diagnosis of GCA

Cut-off values, which maximized both the sensitivity and specificity of a positive test, were defined from sensitivity-specificity curves over the range of observed values (Fig. 3). The cut-off values for each test were comparable whether determined for biopsy negative GCA, biopsy positive GCA or all GCA diagnoses (Table 1). Accordingly, cut-off values to define a positive test were determined as 50 mm/hour for ESR, which is identical to the recommended cut-off in the ACR 1990 Classification Criteria for GCA [6], 20 mg/L for CRP and 300 × 10^9^/L for platelet counts. Based on these cut-off values, the three tests identified a similar proportion of positive results (between 50 and 54%, Table 1, *p* = 0.30). Sensitivity estimates for these three tests were comparable (*p* = 0.45) and ranged between 66% for ESR and 71% for platelets (Table 1). Specificity estimates were also comparable (*p* = 0.11) and ranged between 57% for ESR and 68% for CRP (Table 1).

While both the ROC-AUC analysis and sensitivity/specificity analysis at optimum cut-off values determined that ESR, CRP and platelet counts are equivalent tests with moderate utility for the diagnosis of GCA, there was in fact, only moderate agreement between the three tests in terms of the individual positive/negative classifications (agreement: 67 95% CI 63, 71; prevalence and bias adjusted kappa: 0.34, 95%CI 0.26, 0.42). Therefore, the three-way relationships between ESR, CRP and platelet positive tests for the prediction of GCA were evaluated by logistic regression (Table 2). While each test is significant in individual univariable regression, the multivariable regression demonstrates that both CRP and platelet count are independent predictors of GCA (*p* <  0.001), whereas the ESR is not (*p* = 0.76). In other words, given CRP and platelet results, the ESR is not informative, and a combination of CRP and platelet results may the most informative for a diagnosis of GCA. If a positive test is considered as either CRP > = 20 or platelets > = 300, then this test has high sensitivity for GCA (87, 95% CI 80, 92, Table 3). Alternatively, if a positive test is considered as both CRP > = 20 and platelets > = 300, then this test has a high specificity for GCA (84, 95% CI 77, 90, Table 3). If both CRP and platelet values are below these thresholds, then this may be a useful test for the exclusion of GCA (negative predictive value 77, 95% CI 66, 86). Conversely, if both CRP and Platelet tests are positive, then this may be a useful test for the diagnosis of GCA (positive predictive value 77, 95% CI 67, 85).

**Table 2:** Logistic regression analysis for the association between positive ESR (mm/hr), CRP (mg/L) and Platelets (10^9^/L) tests and GCA. Each predictor is highly significant in individual, univariable regression. However, in the multivariable regression with all three predictors, both CRP and Platelets are independent predictors of GCA (*p* <  0.001), whereas the ESR is not (*p* = 0.76)

ESR > =50	CRP > =20	Platelets> = 300	Odds Ratio (95% CI)	*P*-value
Univariable analysis				
Pos			2.6 (1.6, 4.2)	< 0.001
	Pos		4.3 (2.6, 7.1)	< 0.001
		Pos	4.1 (2.5, 6.9)	< 0.001
Multivariable analysis				
Neg	Neg	Neg	1	
Pos	Neg	Neg	0.8 (0.2, 2.8)	0.73
Neg	Pos	Neg	2.4 (0.7, 8.1)	0.16
Neg	Neg	Pos	2.9 (1.2, 7)	0.020
Pos	Pos	Neg	4.0 (1.5, 10.2)	0.004
Pos	Neg	Pos	3.9 (1.4, 11.4)	0.012
Neg	Pos	Pos	11.8 (2.9, 48.3)	0.001
Pos	Pos	Pos	10.7 (4.8, 23.8)	< 0.001

**Table 3:** Diagnostic performance of a combination of CRP (mg/L) and Platelet (10^9^/L) tests for GCA

Diagnostic Performance	Test Criteria	
	CRP > = 20 *or* Platelets > = 300	CRP > = 20 *and* Platelets > = 300
Sensitivity%	87.1 (80.3, 92.1)	51.1 (42.5, 59.6)
Specificity%	46.6 (37.8, 55.5)	84.0 (76.5, 89.8)
PPV%	63.4 (56.1, 70.2)	77.2 (67.2, 85.3)
NPV%	77.2 (66.4, 85.9)	61.8 (54.2, 69.0)
% Correct	67.4	67.4

## Discussion

While a positive TAB is the gold standard for a diagnosis of GCA, its sensitivity ranges from ~ 70 to > 90%, which underscores that a negative biopsy does not exclude the diagnosis of GCA [9]. This sensitivity rates may be even lower in the large-vessel phenotype of GCA with reported rates being as low as 52%. [10] Skip lesions may contribute to a negative TAB in the presence of GCA; as well in patients with predominant large vessel disease [11]. Therefore, there has been a longstanding interest in the search for serological markers to better aid the diagnosis of GCA with a focus on inflammatory markers [11–21]. In this study we have confirmed that ESR, CRP and platelet counts each have moderate diagnostic utility for a subsequent clinical diagnosis of GCA in the most relevant context, which is all patients referred for a TAB. Further, we have estimated cut-off values for the interpretation of test results. These cut-off values were estimated at 50 mm/hr. for ESR, 20 mg/L for CRP and 300 × 10^9^/L for platelet counts. Importantly, we found no difference in these optimum cut-off values between TAB positive and negative GCA patients, as these tests are likely to be the most useful in TAB negative patients.

The findings of our study are broadly consistent with findings of multiple previous studies, yet direct comparisons are complicated by differences in patients and control definition, and particularly, cut-off values used to define a positive test. Our study identified a cut-off of 50 mm/hr. for the ESR, which is the same as that used in the ACR Classification Criteria for GCA [6], and which has been utilised by a number of similar studies [14, 16, 17]. In comparison, other studies have utilized the upper limit of the normal laboratory range [12, 19], which is substantially lower than either CRP or ESR levels generally seen in GCA. Overall, there has been limited research on appropriate cut-off criteria for interpretation of a positive test for GCA. Importantly, the cut-off values derived from our study for ESR and CRP are comparable to those derived by Heyreh et al [18] who identified a cut-off of 47 mm/hr. for ESR and 24.5 mg/L for CRP, and also similar to those derived by Kermani et al [12] who identified a cut-off of 56 mm/hr. for ESR and 26.9 mg/L for CRP. Studies evaluating platelet count for the diagnosis of GCA have generally utilized a value of 400 × 10^9^/L, derived from laboratory estimates of the normal range [15, 17, 19], whereas, in contrast to ESR and CRP, our estimated cut-off for platelets was within the normal laboratory range.

Studies which report AUC estimates for ESR, CRP and platelets can be directly compared to our study because these are independent of the cut-off values used. We reported an AUC for the ESR of 0.65 (95% CI 0.57, 0.72), and previous point estimates of 0.62 [15], 0.67 [17], 0.59 [19] and 0.71 [21] from four previous studies are within the confidence intervals of our estimate. Our AUC estimate for CRP, 0.72 (95% CI 0.65, 0.79), although identical to one previous study [15], was higher than in two other previous studies, 0.63 [17] and 0.61 [21] respectively. Similarly, our AUC estimate for platelets, 0.72 (95% CI 0.65, 0.79) was virtually identical to the point estimate from three previous studies [15, 17, 19], but higher than a fourth (0.63) [21]. An important caveat for the comparison of these studies to ours is that these previous studies all compared the TAB positive patients to TAB negative patients, which most likely included some TAB negative GCA patients. Regardless, all studies suggest that ESR, CRP and platelets have, at best, moderate ability to distinguish between GCA and non-GCA patients, and as demonstrated by Toren et al [21], the utility of these three diagnostic tests in predicting positive biopsy is decreased for patients who have been initiated on glucocorticoids at the time of referral for biopsy.

Discordance between positive ESR and CRP results is a recognised phenomenon and an evaluation of discordant ESR/CRP laboratory tests in adults indicated clinical differences, with infections, myocardial infarction and venous thrombosis more prevalent in the high CRP/low ESR group, and connective tissue disease, ischemic strokes and transient ischemic attacks more prevalent in the high ESR/low CRP group [22]. This discordance is also observed in GCA, with one study reporting that the CRP has a significantly better sensitivity for GCA compared to the ESR [13]. In our study, this discordance also extended to positive platelet count results, with a kappa agreement between the three tests of only 67%. Although there was a trend for a lower AUC and lower specificity for the ESR test compared to the CRP and platelet tests in our study, this did not reach statistical significance, and we conclude that the tests are in fact comparable at the cut-off values used. It is also quite possible that discordant results may reflect underlying meaningful clinical differences between GCA patients, although this remains to be properly evaluated.

The discordance between ESR, CRP and platelet results in GCA suggest the possibility that a combination of tests may provide the best utility for the diagnosis of GCA. In our study, a multivariable analysis indicated that, given CRP and platelet results, the ESR was essentially redundant, and that specific combinations of CRP and platelet results resulted in high sensitivity and specificity for GCA. Of the three previous studies which evaluated ESR, CRP and platelets by multivariable regression, two concluded, as in our study, that CRP and platelets were the best predictors of GCA [15, 20], whereas the other concluded that ESR and platelets were the best predictors [17]. This latter study also included other blood count markers such as neutrophil: lymphocyte ratio, and monocyte: lymphocyte ratio which, in addition to CRP, which did not reach statistical significance in multivariable regression.

A strength of our study was that it consisted of a state-wide cohort of patients from 5 tertiary referral and peripheral centres hence capturing the full spectrum of patients. Our study included not only patients with a positive TAB but included patients with a clinical diagnosis of GCA, despite having a negative TAB. This is crucial as studies have shown TAB results do not affect the management of patients with suspected GCA [14] and as the ACR Classification criteria were not designed as diagnostic criteria, patients may still be diagnosed with GCA on clinical grounds, especially if there is a good response to glucocorticoid therapy. Hence, we believe our findings more accurately reflect real-world clinical practice. Limitations of this study were that a third of the study population was excluded due to missing data, however the excluded patients had similar age and gender distribution to the included cohort. Further data on concomitant steroid treatment was not available.

## Conclusion

In conclusion, our study demonstrates that ESR, CRP and platelets are moderate but equivalent stand-alone diagnostic tests for GCA, and a combination of CRP and platelets test may provide the most diagnostic utility. For patients with a negative temporal artery biopsy, clinical assessment remains a mainstay of diagnosis.

## Abbreviations

CRP: C- Reactive Protein
ESR: Erythrocyte sedimentation rate
GCA: Giant cell arteritis
ROC-AUC: Receiver Operating Characteristic Area Under the Curve
